# Causes of Sow Mortality and Risks to Post-Mortem Findings in a Brazilian Intensive Swine Production System

**DOI:** 10.3390/ani12141804

**Published:** 2022-07-14

**Authors:** Matheus Saliba Monteiro, Débora Novais Matias, André Pegoraro Poor, Maurício Cabral Dutra, Luisa Zanolli Moreno, Beatriz Martins Parra, Ana Paula Santos Silva, Carlos Emílio Cabrera Matajira, Vasco Túlio de Moura Gomes, Mikaela Renata Funada Barbosa, Maria Inês Zanoli Sato, Andrea Micke Moreno

**Affiliations:** 1 Department of Preventive Veterinary Medicine and Animal Health, School of Veterinary Medicine and Animal Science, University of São Paulo, Av. Prof. Dr. Orlando Marques de Paiva, 87, São Paulo 05508-270, SP, Brazil; matheus.saliba.monteiro@usp.br (M.S.M.); andrepegoraro21@gmail.com (A.P.P.); maucdutra@hotmail.com (M.C.D.); luzanolli@gmail.com (L.Z.M.); bmartinsparra@gmail.com (B.M.P.); anapaula_silva2006@yahoo.com.br (A.P.S.S.); k.rlos89.cabrera@gmail.com (C.E.C.M.); gomesvtm@gmail.com (V.T.d.M.G.); 2 Department of Veterinary Medicine, School of Animal Science and Veterinary Medicine, Federal University of Lavras, Lavras 37200-000, MG, Brazil; deboranovais.matias@gmail.com; 3 Phibro Animal Health Corporation–Av. Pres. Tancredo de Almeida Neves, 1063, São Paulo 071112-070, SP, Brazil; 4 Faculty of Basic Science, Universidad Santiago de Cali, Calle 5 #62-00, Cali 4102, Colombia; 5 Environmental Company of the State of São Paulo (CETESB), Av. Prof. Frederico Hermann Júnior 345, São Paulo 05459-900, SP, Brazil; mrbarbosa@sp.gov.br (M.R.F.B.); misato@sp.gov.br (M.I.Z.S.)

**Keywords:** swine, peripartum, cystitis, palpation, prolapses, obstetric manual intervention, gastric ulcer, arthritis, bacteriological investigation, MALDI-TOF

## Abstract

**Simple Summary:**

Genetic selection increased sow productivity over the last decades, but also increased sow mortality. The impact of mortality includes financial losses, affects labor morale, and raises ethical and animal-welfare concerns. Despite the impact of mortality, the literature is still scarce regarding risk factors for post-mortem findings and bacterial agents involved. Therefore, the present study was conducted to investigate the cause and risk factors related to post-mortem findings in sow death by performing post-mortem examinations, bacteriological investigations, and evaluations of individual sow records. Spontaneous death occurred mainly in the peripartum period. Deaths in the peripartum period were associated with heart failures, genitourinary disorders, and prolapses. Euthanized sows had more locomotor disorders. Sow mortality had a multifactorial etiology including infectious and non-infectious agents; in 75% of the deaths, lesions affecting more than one system were observed. Infections were polymicrobial. The high index of secondary lesions such as gastric ulcers, cystitis, locomotor disorders, and lung pleurisy raise concerns about sow welfare. This study can be used to identify females with a higher risk of injuries and to guide management practices to reduce sow death and increase sow welfare in breeding herds.

**Abstract:**

The present study was conducted to investigate the risk factors for post-mortem findings and causes of sow mortality. A post-mortem examination and microbiological investigation were conducted on 123 sows from a breeding herd with 15,000 dams. The mortality of spontaneous death in sows occurred mostly in the peripartum period (53%; *p* < 0.05). The spontaneous deaths were associated with heart failures, hemorrhagic and perforating gastric ulcers, and liver torsion, while in the euthanized sows, the post-mortem findings were associated with locomotor disorders. A higher body condition score (BCS ≥ 3.5) increased (*p* < 0.05) heart failure on the post-mortem examination. The excessive use of manual obstetric interventions increased sow deaths resulting from cervix/uterus ruptures and increased the odds of death (*p* < 0.05) due to metritis. Sow mortality had a multifactorial etiology. Infections were polymicrobial. The main microbial agents identified from a septic lesion in locomotor, genitourinary, and respiratory systems were *Trueperella pyogenes*, *Escherichia coli*, and *Actinobacillus pleuropneumoniae*, respectively. In conclusion, sow mortality involved multiple risk factors and several bacterial agents. These results indicate that better management practices can reduce sow mortality in swine production and increase sow welfare.

## 1. Introduction

The productivity of sows has changed dramatically during the last decades. Continuous genetic selection led to the high prolificacy of sows and the production of highly lean progeny [1]; it is no longer unusual to observe herds with more than 30 piglets weaned per sow per year. Despite genetic and productivity advances, recent data indicate that sow mortality increased in the last years [2,3,4,5,6], with rates higher than 10% [5,7,8,9]. Sow mortality and longevity have a great economic impact and are concerns for animal welfare [10,11,12].

Sow mortality increases the direct costs related to the replacement of gilts and increases non-productive days, the use of medication, and the slaughterhouse carcass condemnation [4,6,8,11,13]. The mortality rate increases the indirect costs due to the partial loss of genetic value, retaining a female that should normally be removed/culled, and increasing the numbers of gilts with less health status adaptation to the farm challenges [14,15,16]. Despite these data, there is high heterogeneity in the studies that evaluated the post-mortem findings. Regarding the methodology to determine the lesions, in some studies, the post-mortem examination was performed by its author [3,8,17], whereas, in others, the results were obtained by data collection [18,19,20]. As stated by Ala-Kurikka et al. [11], some studies are retrospective analyses of field data and the post-mortem examination protocols are not well detailed. Furthermore, it was described that in fewer than 50% of the post-mortem examinations, the farmers’ impressions about the cause of death are partly correct [11]. These reports indicate that neither farmers nor veterinarians have a real understanding of the post-mortem lesions and their impact on sow mortality. Regarding this, the studies based on sow mortality did not conduct in-depth investigations of pathogens. Studies that performed a microbiological assay only conducted this analysis on a few sows and did not describe the methods in detail [7,8,20]. The little information about bacterial agents enrolled in the infectious causes of a sow’s death makes preventive actions based on microbiological epidemiology difficult [21].

The economic losses for sow mortality demonstrate that it is possible to increase the productivity with just corrections of the risk factor for sow mortality. The mortality rate reduction will have benefits for animal-welfare improvement. Therefore, it is pivotal to identify the causes of mortality and their bacterial agents and the risk factors aiming to reduce the sow mortality. Thus, the present study aimed to present the post-mortem findings as well as some of their risk factors and to conduct a microbiological assay based on technologies such as matrix assisted laser desorption/ionization time-of-flight (MALDI TOF) and polymerase chain reaction (PCR) to characterize the pathogen richness associations related to sow mortality.

## 2. Materials and Methods

### 2.1. Ethics Committee Approval

The Ethics Committee for the Use of Animals of the School of Veterinary Medicine and Animal Science of the University of São Paulo approved this experiment under protocol 9465041119. All animal procedures were performed according to legal and ethical standards.

### 2.2. Animal and Herds

This study was conducted in a breeding herd with 15,000 sows located in the Brazilian Midwest (Mato Grosso State). The herd genetic line was commercially crossbred (Large White, Landrace, and Duroc). The estrous detection was conducted twice a day (8:00 a.m. and 4:00 p.m.) using a mature boar. After estrous detections, the artificial insemination was conducted at time 0 and each 24 h until estrous finishing. The artificial insemination method was post-cervical (PCAI). A pregnancy check was performed three times with an ultrasound scan at 4, 8, and 12 weeks after mating. The pre-mating gilts were kept in a group of 20 animals. After mating, the gilts/sows were moved to a gestation crate and then to group housing (six sows per group) after the first or double pregnancy check. The transference from gestation to the farrowing room was conducted 5–7 days before the expected farrowing day (D0 = first mating). During the lactation period, the sows were kept in farrowing crates. The peripartum period was defined as the period from 7 days before farrowing until 7 days post-farrowing. Throughout the experimental period, the sows were kept on solid/concrete and partially slatted floors and had free access to water. Piglets were weaned at 24 days of age. The all-in/all-out batching period was 3 to 5 days. Sows received water in common water troughs or via nipple and feed via drop feeders. Sow mortality was calculated based on the total number of female deaths (i.e., spontaneous deaths and euthanized sows) that occurred in the herd per total number of sows in the breeding herd inventory. Sudden deaths and sows observed with a previous health condition and that died were both classified in the spontaneous sow death group. When sows were unable to be moved to the abattoir due to health conditions, euthanasia was conducted by the farm personnel and this death was accounted for in the mortality rate. Sows with locomotor disorders that were not able to stand up voluntarily were euthanized. Due to government regulation, prolapsed sows cannot be sent to abattoirs; therefore, these sows were euthanized at the farm. Females sent to abattoirs were not accounted for in the mortality rate calculation. The sow annual herd mortality rate was 12.5% in the last 12 months, and the replacement rate was 50%.

The herd was free of PRRSV and *Brachyspira hyodysenteriae* and positive to *Mycoplasma hyopneumoniae*, PCV-2, influenza, and *Actinobacillus pleuropneumoniae*. The replacement program from the commercially genetic line was internal, and only purebred gilts from nucleus herds were externally replaced. The females were vaccinated during the nursery period against *M. hyopneumoniae*, PCV-2, and *Glaesserella parasuis* every 6 months. The vaccination against *A. pleuropneumoniae* and *Lawsonia intracellularis* was conducted in the nursery period. Gilts and sows were vaccinated against *Erysipelohthrix rhusiopathiae*, parvovirus, and *Leptospira interrogans* (ser. Bratislava, Pomona, Icterohaemorrhagiae, Canicola, Grippotyphosa, and Hardjo).

During gestation, the females were fed with 2.5 kg once a day with the help of automatic drop feeders. After transference to the farrowing room, the sows were manually fed with 1.0 kg three times a day (3.0 kg total). During lactation, the dams were manually fed ad libitum and the feed was only provided when the feeding trough was empty. The nutritional mash feed energy levels, crude protein, and crude fiber were as follows: gestation feed, 2890 Kcal/Kg, 14.9%, and 7.2%, respectively; peripartum feed, 3083 Kcal/Kg, 19.3%, and 5.2%, respectively; and lactation feed, 3475 Kcal/Kg, 16.9%, and 3.3%, respectively.

### 2.3. Post-Mortem Examination and Sample Collection

A total of 100 females that had spontaneous deaths and 23 that were euthanized were necropsied; the post-mortem examination was conducted by two veterinarians.

The lesions were classified according to the organic system as follows: locomotor disorders: arthritis, suppurative myositis, fractures, non-infectious locomotor disorders, and osteomyelitis; gastric disorders: gastric ulcer, hepatic lobe torsion and rupture, rectal prolapse, splenomegaly, spleen torsion or rupture, mesentery torsion, stomach dilatation, hepatomegaly, and intestinal intussusception; genital disorders: endometritis, the presence of a fetus or placenta retention, uterine prolapse, cervix rupture, uterine hemorrhage, and dystocia; respiratory disorders: pleurisy, pulmonary edema, pulmonary congestion, pulmonary consolidation, pulmonary abscess, and pleural abscess; systemic disorders: peritonitis, polyserositis, and splenic abscess; and urinary disorders: cystitis, pyelonephritis, hydronephrosis, and bladder prolapse.

Different types of prolapse were grouped together to observe if the peripartum period increased their risk of occurrence. Locomotor disorders and prolapses can lead to deaths due to secondary causes such as sepsis and circulatory shock; however, in these cases, these secondary diagnoses were not accounted for since they were directly the consequence of the primary cause. Heart failures were diagnosed when excessive pulmonary edema and congestion occurred and were associated with excessive cavitary fluids (hydrothorax, ascites, and hydropericardium).

Diagnoses of septicemia were only considered if it was the primary cause of death and not as a consequence of arthritis, prolapses, pyelonephritis, metritis, and cystitis. Polyserositis and mastitis diagnoses were based only on a visual approach.

Dead sows were examined within 6 h after death. Information regarding the parity order, gestational phase, observed clinical symptoms, and recent medical treatment history was collected. The external examination evaluated the body score condition [22] and claw injuries (adapted from Deen et al. [23]). The cranial cavity was not opened unless a history of nervous signs accompanied the death. When gross lesions suggested an etiologic agent, samples were collected for microbiological evaluation and the pleurisy evaluation was based on Merialdi et al. [24]. The methods for pneumonia and gastric ulcer evaluation were adapted from Christensen et al. [25] and Gottardo et al. [26], respectively. For suspected cases of cystitis, urine was collected with a sterile needle and syringe, as well as swabs kept in a Stuart transport medium from the mucosal wall of the urinary bladder. For metritis and pyelonephritis, a swab was collected and kept in a Stuart transport medium. For muscular abscesses/suppurative myositis and arthritis, the sample was collected with a sterile needle and syringe or a swab and kept in a Stuart transport medium. When gross lung lesions were observed, the affected lobes were collected and placed in sterile bags.

The samples collected were immediately refrigerated (5–8 °C) and sent to the Laboratory of Swine Health at the Faculty of Medicine Veterinary and Animal Science, University of São Paulo (FMVZ-USP). All samples were submitted for microbiological isolation and identification. Lung samples were also submitted for PCR assay.

### 2.4. Bacterial Isolation

The samples collected were plated in MacConkey agar (Difco, Detroit, MI, USA), Chromagar Orientation™(Difco), Columbia blood agar with 5% sheep blood (with and without a streak of *Staphylococcus aureus*), and Brucella agar with 5% sheep blood, hemin, and vitamin K1. The agar plates were incubated under aerobic, microaerophilic, and anaerobic conditions for 24–48 h at 37 °C. After microbiological isolation, the colonies were incubated in BHI broth (Brain Heart Infusion, Difco) and screened by matrix assisted laser desorption/ionization time-of-flight mass spectrometry (MALDI-TOF MS).

### 2.5. Bacterial Identification

The selected colonies were identified by MALDI-TOF MS, and a bacterial culture was subjected to ribosomal protein extraction using the protocol described by Hijazin et al. [27]. Mass spectra were acquired using a Microflex™ mass spectrometer (Bruker Daltonics, Billerica, MA, USA) and α-cyano matrix (10 mg α-cyano-4-hydroxycinnamic acid mL^−1^ in 50% acetonitrile/2.5% trifluoroacetic acid). Each strain was distributed in three wells; for each plate, two readings were performed with the FlexControl™ software (Bruker Daltonics, Billerica, MA, USA) using the MTB_autoX method. Finally, the BioTyper™ 3.0 software (Bruker Daltonics) compared the captured spectra for each strain with the manufacturer’s library for bacterial identification. Standard Bruker interpretative criteria were applied; scores ≥ 2.0 were accepted for species assignment, and scores ≥ 1.7 but ≤2.0 were accepted for genus identification. 

### 2.6. DNA Extraction, PCR Assay, and Electrophoresis

Lung samples were submitted for DNA extraction according to Boom et al.’s [28] protocol with the previous enzymatic treatment of 60 min at 37 °C, with 100 mg of lysozyme and 20 mg of proteinase K (United States Biological, Swampscott, MA, USA). Samples were stored at −20 °C until processing.

Polymerase chain reaction (50 µL) used 5 µL of genomic DNA, ultrapure water, 10X PCR buffer, 1.5 mM MgCl_2_, 200 µM of dNTPs, 10 pmol of each primer, and 1.25 U of Taq-DNA-polymerase (Fermentas Inc., Glen Burnie, MD, USA). Primers and reaction parameters were adjusted for each agent: *Mycoplasma hyopneumoniae* [29], *Bordetella bronchiseptica* [30], *Actinobacillus pleuropneumoniae* [31], *Glaesserella parasuis* [32], *Pasteurella multocida* [33], PCV-2 [34], *Streptococcus suis* [35], and *Trueperella pyogenes* [36]. The amplified products were stained with BlueGreen^®^ (LGC Biotecnologia, São Paulo, Brazil) and separated by electrophoresis in 1.5% agarose gels, using the 100 bp DNA Ladder^®^ (New England Biolabs Inc., Ipswich, MA, USA).

### 2.7. Statistical Analysis

The descriptive analysis from zootechnical data and gross lesions was performed utilizing the Rstudio 1.4v and GraphPad Prism 7. The gross lesions’ associations and bacterial agent associations were obtained by the Complex Heatmap package (Rstudio 1.4v) [37]. The results were presented in contingency tables. The *p*-value was obtained by generalized mixed effects fitted by binomial distribution. It considered the parity order, type of death (spontaneous or euthanasia), and body score condition as random effects to observe the effects of the peripartum period on post-mortem findings, the impact of cystitis in pyelonephritis, and the impact of manual obstetric interventions in metritis. To observe the impact of the body score condition on the post-mortem findings, the model included parity order, gestational phase, and type of death as random effects. The odds ratio (OR) was presented in a confidence interval (CI) of 95% (OR; CI: MIN–MAX). For claws’ injuries, the residue normality and homogeneity of variances were verified, and, when necessary, a logarithm or Box-Cox transformed the data. If, after the transformation, the data did not follow the parametric premises, the data were submitted for non-parametric tests (Kruskal–Wallis and Friedmann tests).

## 3. Results

### 3.1. Parity Order, Body Condition Score (BCS), and Productive Stage

**Figure 1:**
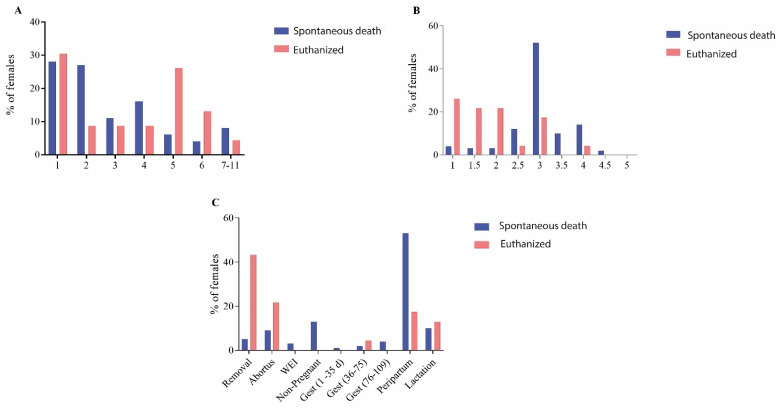
Characteristics of spontaneous death and euthanized females. (**A**) Parity order; (**B**) body condition score; (**C**) productive stage. Removal: females that were moved to gestation rooms destined for removal but died before being moved to the slaughterhouse; Abortion: pregnant females that aborted in the last 7 days before death; WEI: females that died during weaning–estrous interval; Non-Pregnant: females that returned to estrous and were not re-inseminated; Gest (1–35 d): 0–35 d of gestation; Gest (36–75 d): 36–75 d of gestation; Gest (76–107): 76–107 d of gestation; Peripartum: 110 d of gestation until 7 days after farrowing; Lactation: 8 d after farrowing until weaning.

The parity order of death was similar (*p* = 0.145) between the euthanized and dead sows (Figure 1A). The body condition score was lower in the euthanized sows than in spontaneously dead ones (average 1.9 vs. 3 *p* < 0.001) (Figure 1B). Regarding the productive stage, a higher mortality rate was observed during the peripartum period in the spontaneous death group than in euthanized sows (53% vs. 13%; *p* < 0.001). Euthanasia occurred for humanitarian reasons in sows that were unable to be sent to the slaughterhouse for some reason, such as an inability to get up, and for prolapses. Females that aborted were predisposed to die (10% of all the spontaneously dead and 20% of the euthanized sows) (Figure 1C). Females that, for some reason, did not get pregnant represented 17% of the deaths in the spontaneous death group of sows. 

### 3.2. Risk Factors for Post-Mortem Findings

The association is presented in Table 1. The peripartum period increased (*p* < 0.05) the odds for post-mortem findings related to heart failure, genital disorders, urinary disorders, and prolapses but did not have an impact (*p* > 0.05) on respiratory disorders, gastric ulcers, and abdominal organs’ torsions. A low body condition score (BCS ≤ 2) was associated with locomotor disorders on the post-mortem examination. A higher body condition score (BCS ≥ 3.5) increased (*p* < 0.05) heart failure on the post-mortem examination. The BCS was not associated (*p* > 0.05) with genitourinary and respiratory disorders on the post-mortem examination. Locomotor members with moderate and severe gross lesions in at least one claw increased (*p* < 0.05) post-mortem findings related to locomotor septic disorders (arthritis and suppurative myositis). The occurrence of cystitis during the post-mortem examination increased (*p* < 0.05) the occurrence of pyelonephritis. Manual obstetric interventions increased (*p* < 0.05) the occurrence of metritis on the post-mortem examination. More (*p* < 0.05) pathological findings were observed related to locomotor disorders in euthanized animals.

### 3.3. Post-Mortem Findings

The post-mortem findings were categorized according to the system and in spontaneous and euthanized deaths (Table 2). Infections per system according to parity order, body score condition, and production phase can be observed in the Supplementary Material (Tables S1–S6). Septic arthritis had a significant impact on mortality, being responsible for 11% and 39.1% of the post-mortem findings in spontaneous death and euthanized females, respectively. Suppurative myositis was diagnosed in 9% and 30.4% of the necropsied females in spontaneous death and euthanized females. Heart failure was only diagnosed in the spontaneous death group, being responsible for 15% of cases in this group. Gastric ulcers were diagnosed in 30 animals (24.4%) and were the main diagnoses for six animals that had hemorrhage and two sows with perforated gastric ulcers. Endometritis had high prevalence in the post-mortem examination, with an occurrence in 22% of the spontaneous death group and 8.7% of the euthanized animals. Pleurisy was the most frequent lesion identified in the post-mortem examination, being reported in more than 50% of the animals examined. Cystitis and pyelonephritis were diagnosed in 40 (32.5%) and 15 (12.2%) animals, respectively. The prolapses were categorized as rectal, uterine, and bladder prolapses. The most frequent was uterine prolapse (seven animals, 5.7%), followed by rectal prolapse (five animals, 4%) and bladder prolapse (two animals, 1.6%). Mastitis presented minimal impact on gross lesions in dead sows in this study.

#### 3.3.1. Locomotor Disorders

Locomotor disorders were more prevalent in euthanized animals. The most common pathological findings were of a septic origin (arthritis and suppurative myositis). Osteomyelitis was associated with septic arthritis.

#### 3.3.2. Gastrointestinal Pathological Findings

During the post-mortem examination, the most prevalent lesions observed were gastric ulcers (30 animals) and abdominal organ torsion. Considering abdominal organs, the most frequent kind of torsion was from the hepatic lobe (five animals), followed by spleen torsion (three animals). Splenomegaly was observed in four animals (Table 2). Other lesions observed were hepatomegaly, intestinal intussusception, mesentery torsion, and stomach dilatation.

From animals presenting gastric ulcers (30/123, 24.4%), hyperkeratosis was observed in 6.7% of the stomachs. In 40% of sows, the lesions affected less than 33% of the pars esophagea. In 20% of these animals, the lesion reached between 33–66% of the pars esophagea; in 6.7% of animals, the lesion affected more than 66% of the pars esophagea. The gastric ulcers’ classification also included hemorrhagic ulcers and perforated gastric ulcers, which were observed in 20% and 6.7% of the females, respectively (Supplementary Material Table S7).

#### 3.3.3. Genital Tract Pathological Findings

The lesion most frequently detected was endometritis (24 animals). Endometritis was observed alone or in association with fetus and placenta retention (Table 2). Uterine prolapses were observed in seven animals (5.7%). Cervix rupture was responsible for 6% of the findings in the spontaneous death group (Table 2).

#### 3.3.4. Mammary Pathological Findings

All the mastitis diagnosed was from chronic processes and had abscess formation as a clinical singularity.

#### 3.3.5. Respiratory Pathological Findings

Lung edema and congestion were often observed. When these lesions were associated with heart failure as the primary diagnosis, respiratory disorders were excluded (Table 2). Lung consolidation areas were observed in 16 animals (13%). Of these, only six animals had more than 10% of their lung area affected. A total of 63 animals had pleurisy, of which 36 (57%) had pleurisy degree 1 and 12 (19%) had pleurisy degree 2. Less than 25% of the females with pleurisy were categorized with pleurisy degree 3 or 4. Lungs with consolidation and pleurisy were collected for microbiological assay. Individual data from lobes’ consolidation and pleurisy degree associated with microbiological identification can be seen in the Supplementary Material (Table S8).

#### 3.3.6. Systemic Pathological Findings

Peritonitis was observed in seven sows associated with abdominal organ ruptures (stomach perforation due to gastric ulcer or cervix rupture due to manual obstetric intervention). Polyserositis had a low occurrence and was identified in four animals (Table 2).

#### 3.3.7. Urinary Pathological Findings

Cystitis was diagnosed in 40 females (32.5%). Pyelonephritis was diagnosed in 15 females. Other, more unusual lesions were hydronephrosis (two animals) and bladder prolapse (two animals) (Table 2).

### 3.4. Pathological Findings Associations

**Figure 2:**
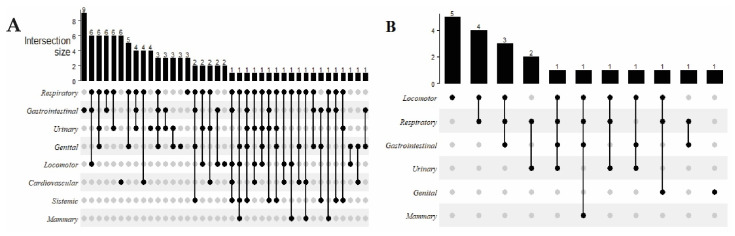
The number of females and post-mortem findings associated with spontaneous death (**A**) and with euthanized females (**B**).

Only 27% of the animals that had spontaneous death presented pathological findings in only one system. A total of 32%, 29%, 9%, and 3% of cases from the spontaneous death group were associated with pathological findings in two, three, four, and five systems. The gastrointestinal system was affected in most cases with single lesions found in the spontaneous death group (Figure 2A). In contrast, in euthanized animals, the system that was associated with the most single lesions found was the locomotor.

### 3.5. Claws’ Injuries

It was observed that 93.5% of the females had lesions in at least one claw. The most common claws’ injuries were one or more toes slightly longer than normal (26% of the claws evaluated) followed by overgrowth or erosion in the soft heel tissue (23.4%) and one or more toes slightly larger than normal (17.5%). Cracks in the wall (vertical, horizontal, and oblique), separation at the juncture (soft heel and wall tissue), and separation along the white line were observed in 11.2%, 10.6% and 8% of the claws, respectively. One or more toes significantly larger or longer than normal were observed in 6.7% and 6.3% of the claws, respectively. Long or larger toes that affect the gait when walking were observed in 3.6%, and 3.4% of the claws, respectively. Injuries in the coronary band were found in 3.9% of the claws. Less common injuries were cracks in the dewclaw, claw necrosis, abscesses, torn claws, and claws partially or completely missing (Supplementary Material Table S9).

**Figure 3:**
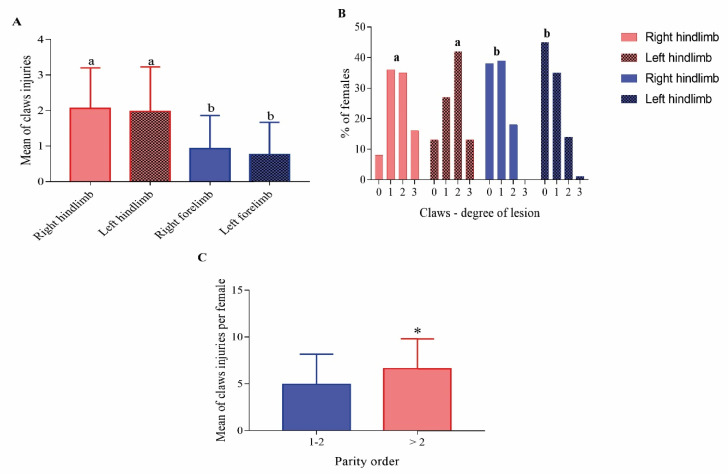
(**A**) Claw injuries according to mean of claws’ injuries in locomotor forelimb and hindlimb; (**B**) claw injuries according to the degree of the lesion in locomotor forelimb and hindlimb; 0: no claws injuries; 1: claws with a discrete lesion; 2: claws with a moderate lesion; 3: claws with a severe lesion; (**C**) claw injuries according to parity order. Within graphics means without a common superscript (a-b or *) differed statistically (*p* < 0.05).

The locomotor forelimb had fewer claws’ injuries (*p* < 0.05) (Figure 3A) and the lesions observed had less impact on the sow’s health (*p* < 0.05) when compared to locomotor hindlimb (Figure 3B). Older parity sows (parity order equal or higher than 2) had more claw injuries (*p* < 0.05) than younger sows (parity 0 and 1) (Figure 3C).

### 3.6. Microbiological Assay

#### 3.6.1. Locomotor Septic Disorders

A total of 20 females had arthritis and 16 had suppurative myositis. One sow had two members with suppurative myositis. Therefore, a total of 17 samples were collected. Of these, 20 females with arthritis, mixed infection were observed in 65% of the samples, being isolated in 25%, 15%, 15%, and 10% of the samples, and two, three, four, and five or more different bacterial species. From the cases of suppurative myositis, in 47% of the samples, only a single bacterial species was identified, while the remaining infections were caused by two (23.5%), three (23.5%), and four (6%) different bacterial species.

From arthritis, a total of 30 different bacterial species were identified, and the most common isolated agent was *Trueperella pyogenes*, being isolated in 75% of the samples. *Acinetobacter lwoffii* was isolated in 20% of the samples, followed by *Actinomyces hyovaginalis*, *Bacteroides pyogenes*, *Corynebacterium amycolatum*, and *Staphylococcus simulans*, all being isolated in 15% of the samples. *Aerococcus viridans*, *Corynebacterium frankenforstense*, *Escherichia coli*, *Staphylococcus cohnii,* and *Staphylococcus hyicus* were isolated in 10% of the samples. The bacteria association is presented in the Supplementary Material (Figure S1). *T. pyogenes* was the single isolated pathogen in 57% of the cases. Other species isolated as single pathogens were *S. hyicus*, *Bacteroides pyogenes*, and *Streptococcus dysgalactiae*.

For the cases of suppurative myositis, a total of 15 different bacterial species were identified, with *T. pyogenes* isolated in 76.5% of the samples. *S. dysgalactiae* was isolated in 17.5% of cases. *A. lwoffii*, *A. viridans*, *B. pyogenes*, and *E. coli* were isolated in 11.8% of the samples. Other pathogens isolated in less than 10% of the samples were *A. hyovaginalis*, *S. haemolyticus*, *S. porcinus*, and *S. hyicus*. The bacteria association is presented in the Supplementary Material (Figure S2). *T. pyogenes* was the single isolated pathogen in 67% of the cases. The other pathogens that were isolated as a single pathogen in myositis were *A. hyovaginalis*, *A. viridans*, and *S. dysgalactiae*.

#### 3.6.2. Genital Tract Infections

The 24 samples collected from the uterus presented macroscopical signs of endometritis. Nineteen bacterial species were isolated, with mixed infections in 45.8% of the samples. *E. coli* was isolated in 62.5%, *S. hyicus* in 12.5%, *E. faecalis* in 12.5%, *B. pyogenes* in 8.3%, *Clostridium perfringens* in 8.3%, *E. faecium* in 8.3%, *Streptococcus orisratti* in 8.3%, *S. suis* in 8.3%, and *A. hyovaginalis* in 8.3% of the samples. The bacteria association is presented in the Supplementary Material (Figure S3). *E. coli* was a single isolated pathogen in 61.5% of the cases. The other pathogens that were isolated as single pathogens were *B. pyogenes*, *S. aureus*, *S. cohnii*, and *S. hyicus*.

#### 3.6.3. Urinary Tract Infections

Microbiological samples were collected from 40 females presenting signs of cystitis and 15 animals with pyelonephritis. In the cystitis cases, 24 bacterial species were isolated, with mixed infections in 45% of the samples; for pyelonephritis, 16 bacterial species were isolated, and mixed infections were observed in 73.3% of the samples. The most frequent agent was *E. coli*, identified in 52.5% and 80% of the samples collected from cystitis and pyelonephritis, respectively. In the cystitis cases, *A. lwoffii* and *P. mirabilis* were isolated, both in 12.9% of the samples; *A. viridans*, *E. faecalis*, *E. faecium*, and *Providencia rettgeri* were isolated in 7.5% of the samples. The pathogen most isolated from single infections was *E. coli* (Supplementary Material Figure S4). In pyelonephritis cases, the most frequent agents after *E. coli* were *Corynebacterium xerosis* and *C. perfringens*, being isolated in 20% and 13.3%, respectively. The only bacterium isolated as a single pathogen in pyelonephritis was *E. coli* (Supplementary Material Figure S5).

#### 3.6.4. Respiratory Tract Infections

Tissue samples were collected from 68 lungs. The pathogen most often identified was *A. pleuropneumoniae* (19.1%), followed by *T. pyogenes* (7.4%), *S. suis* (5.9%), *P. multocida* (5.9%), and *M. hyopneumoniae* (1.5%). All samples were negative for PCV-2 and *B. bronchiseptica*. Approximately two-thirds of the lungs (68%) were negative for pathogen detection by culture and PCR.

## 4. Discussion

The peripartum period was the most critical physiological phase for sow mortality and accounted for 53% of the deaths in the spontaneous death group. Other studies also described that the peripartum phase represents more than 50% of all female deaths [9,38]. Herds with high sow mortality and poor health conditions have difficulty in guiding removal practices based only on productivity due to a reduction in gilt pool size [38]. In this scenario, females that normally should be culled remain for long periods in the herds, increasing sanitary risks [14]. In fact, in the studied herd, the mortality is higher than 10%, which is considered to be higher than the desired amount.

Deaths that occurred during the peripartum period had more post-mortem findings related to heart failures, genitourinary lesions, and prolapses. Stressful events (heat stress, transport, fighting) and a higher average body weight contribute to increasing mortality during the peripartum period due to heart failures [39]. Management related to farrowing is important during the peripartum period. The transport of females to the farrowing room should be performed in the coolest times of the working day. Although in the present study the room temperature was not evaluated, the farm was located in the Brazilian Midwest, where hot temperatures (over 30 °C) are common during the spring and summer. This fact, associated with female transference to farrowing rooms throughout the day, could contribute to increasing mortality during the peripartum period, especially in sows with body score conditions higher than 3.5 due to the risk of heart failure. Despite this fact, the deaths with heart failures in the present study were in the range of other studies in the literature that reported between 10% [17] and 30% [18,20] of deaths due to this cause.

Manual obstetric interventions should be carefully applied and only when necessary [40,41]. It was observed that manual obstetric interventions led directly to the mortality of 9% of the sows in the spontaneous group due to uterine ruptures/hemorrhages and led indirectly to mortality due to an increase in the odds of endometritis development when compared to sows that died and were not exposed to birth assistance. Manual farrowing assistance should be performed in around 10% of the females [42]; however, on commercial farms, it can be as high as 60% [40].

There are different etiologies for prolapses, although there is little literature information indicating which prolapse type is more common in females [43]. The peripartum period is a stage in the female productive cycle that increases the risk for prolapse occurrence due to increased abdominal pressure and due to cervix and pelvis opening and relaxation [43,44,45]. Moreover, during the peripartum period, it is described the sphincter relaxation, including the bladder/urethra sphincter, which can contribute to the passage of the bladder through urethral canal. Despite this, the studies on sows are scarce and the risk factors are poorly understood, especially for bladder prolapses. To the best of our knowledge, this is the first study that described bladder prolapses in sows.

For the other types of prolapses, different authors reported that rectal prolapses occur mainly in females of first parity, due to muscular immaturity [44], while uterine and vaginal prolapses occur mainly in older parity sows, as a consequence of a possible loss of uterine tonus [43]. In fact, in our study, we observed that, from the seven females that had uterine prolapse, six belonged to parity higher than 3; whereas, from rectal prolapses, of the five female necropsies, three were parity 1.

Similarly to our study, different authors describe locomotor disorders as an important post-mortem finding in sows [3,7,16]. Studies that evaluated claws reported that more than 90% of the sows had an injury in at least one claw [46]. We identified that the most common lesions observed were toes that were longer or larger than normal, overgrowth or erosion in the soft heel tissue, cracks in the wall, and separation at the juncture (soft heel and wall tissue) and along the white line. Similar lesions were reported in another study [47]. In our study, claws with injuries that moderately or severely impacted the sow’s health increased the odds for infectious locomotor disorders in sow deaths. These data indicate that claws’ injuries have a high prevalence in the sow herds, impair animal welfare, and can contribute to increasing sow mortality.

Hemorrhagic and perforated gastric ulcers and lobe hepatic torsion are conditions responsible for sudden deaths. Some studies described that feed supply once a day in automatized feeders is a risk factor for both gastric ulcers and abdominal organ torsions when compared to feed supply two or three times a day [48,49]. In fact, in the studied herd, sows were fed only once a day during the gestational period. However, due to the study characteristics, it is not possible to make more assertions. It is important to emphasize that a quarter of the sows necropsied had some degree of gastric ulcer, indicating the impact of these lesions on the sow herds. Despite these values, these data are consistent with other studies [3,7,8,17]. A higher occurrence of gastric ulcers can also indicate problems with animal welfare [26,50].

Respiratory tract infections had a high prevalence in this study, especially pleurisy. However, the lung gross lesions were in the vast majority non-exudative pleurisy degree 1; consolidations affected only a few animals, with gray aspect and in less than 10% of the lung area. These conditions can indicate that these lesions have a chronic aspect; due to this, two-thirds of the lungs were negative for the investigated pathogens. Even in acute infection, lungs can be PCR negative [51] for chronic lesion; this index is even higher since the infection could be cleared [52,53]. Despite the negative results, our data show that some pathogens could impact the females’ health. *A. pleuropneumoniae* was the most often identified pathogen in the lungs and was associated with severe pleuropneumonia. Some strains of this pathogen are highly virulent and can lead to sudden death [54]. Other pathogens identified were *P. multocida*, *S. suis*, and *T. pyogenes*. Recent studies indicate that *P. multocida* is associated with pleurisy; *T. pyogenes* is associated with a thrombus–embolic abscess in the lungs after infections in distinct body parts [55].

Despite the differences regarding the infection site and the prevalence of arthritis and suppurative myositis, both diseases have similar pathogens as etiologic agents. *T. pyogenes* was the bacterium most frequently isolated, followed by *S. dysgalactiae* and *S. hyicus*. Similar results obtained from dead sows were found by other authors [3,7]. However, these studies isolated septic locomotor disorders of only a few animals and did not clearly describe the methodology for the isolation and identification, nor did they describe possible microbial associations. *T. pyogenes* was already isolated in arthritis [56]; however, to our knowledge, there are no studies that described *T. pyogenes* isolation from suppurative myositis in swine. *T. pyogenes*, *S. dysgalactiae*, and *S. hyicus* are present in skin and mucosal microbiota [56]; repeated injections under poor hygiene conditions can facilitate the penetration and migration of these pathogens to inactive immune body sites and can facilitate the establishment of the infection [57].

The genitourinary infection has a complex and polymicrobial etiology [58,59]. Moreno et al. [58] described that the majority of the sows with cystitis had mixed infections, including *Aerococcus*, *Corynebacterium*, *Staphylococcus*, *Streptococcus,* and *Enterococcus*. Comparable results were observed in the present study. Studies by Moreno et al. [58], Poor et al. [59], and this one used a similar technique for bacterial identification (MALDI-TOF MS); however, Moreno et al. [58] collected the urine during spontaneous micturition, and Poor et al. [59] collected samples from the vagina with the use of a speculum and swab. In the present study, urine and uterine samples were collected directly from the bladder and uterus during a post-mortem examination, reducing contaminants and indicating pathogens with the ability to colonize the sow bladder and uterus. It was probably a collection directly in the site of the infection that reduced urethra and vaginal microbial species; due to this, mixed bacterial isolates were reduced when compared to Poor et al. [59] and Moreno et al. [58]. Thus, this study may indicate the most relevant pathogens for cystitis and metritis development. For pyelonephritis, the most common pathogen isolated was *E. coli*, indicating an ascending infection; however, this was not the only bacterium isolated from the kidneys. The occurrence of *Staphylococcus* (*S. sciuri*, *S. saprophyticus*, *S. warneri*, and *S. cohnii*) and *C. xerosis* indicates that these pathogens could have virulence factors to evade the immune system and establish infection. To our knowledge, no study identified pathogens enrolled in the genitourinary infection in sows from samples collected directly from the bladder and uterus and submitted for more recent microbiological techniques such as MALDI-TOF.

In previous studies, biochemistry methods identified *E. coli*, *Streptococcus*, and *Staphylococcus* genera from metritis [60]. The use of MALDI-TOF in our study allowed a detailed identification of the most common associations observed in metritis cases: *E. coli* with *Enterococcus* genera, *E. coli* with *S. suis* and *S. orisratti*, and *E. coli* with *S. hyicus*. However, regarding genital infection, in the present study it was observed that *Corynebacterium* genera and *B. pyogenes* may have a key role in the development of metritis. These pathogens were already identified as relevant for the development of disease in sows in other studies [59,61].

The use of MALDI-TOF MS has been very useful to identify less frequent and rarer bacterial species that can be underestimated using traditional biochemistry methods [59,62,63,64,65]. Based on this, the MALDI-TOF can be a valuable tool to describe the richness of the pathogens’ enrolled in the development of diseases and deaths in sows. Despite the isolation of mixed bacterial species in some lesions, some pathogens may be more important for the onset or development of the disease.

Limitations of the present study are the lack of detection of some viruses, such as the influenza virus, which can negatively impact the respiratory conditions described. Another point is that some bacterial agents may not grow on the culture media used; therefore, some pathogens may not have been identified. Although carried out in only one intensive swine production system, the breeding population was of considerable size and the management practices adopted were similar to those used in most herds in the world. Despite this, the mortality rate of sows may change according to the herd health status in different countries or region; one example, is that the studied population was PRRSV free.

## 5. Conclusions

Sow mortality is multifactorial and post-mortem findings are associated with several risk factors. The occurrence of pathological findings in more than one system was a common finding. It was observed that some pathogens play a major role in the development of diseases, contributing to sow mortality, namely, *A. pleuropneumoniae* and *T. pyogenes*, in the respiratory tract. *T. pyogenes* followed by *S. dysgalactiae* and *S. hyicus* are the main pathogens involved in arthritis and suppurative myositis, and *E. coli* for genitourinary infections. The higher prevalence of claw injuries, cystitis, and gastric ulcers raises serious concerns about animal welfare and sow mortality. The high incidence of endometritis and cervix rupture observed indicates that manual obstetric intervention has not been applied judiciously.

## Figures and Tables

**Table 1:** Risk factors for post-mortem findings.

**Post-Mortem Findings**	Deaths on Peripartum * **(N = 56)**	**Deaths out of** Peripartum * **(N = 67)**	***p*-Value**	**OR (CI)**
Heart failure				
Present (N = 15)	12	3	0.036	5.73 (1.43–33.54)
Absent (N = 108)	44	64		
Genital disorders				
Present (N = 37)	28	9	0.007	6.33 (2.5–17.44)
Absent (N = 86)	28	58		
Urinary disorders				
Present (N = 44)	27	17	0.035	2.71 (1.19–6.29)
Absent (N = 79)	29	50		
Prolapses				
Present (N = 13)	12	1	0.001	17.39 (2.41–141.27)
Absent (N = 110)	44	66		
Respiratory disorders				
Present (N = 78)	39	39	0.725	1.64 (0.73–3.74)
Absent (N = 45)	17	28		
Abdominal organs’ torsion				
Present (N = 12)	3	9	0.329	0.36 (0.06–1.57)
Absent (N = 111)	53	58		
Gastric ulcers				
Present (N = 30)	11	19	0.224	0.61 (0.23–1.54)
Absent (N = 93)	45	48		
**Post-mortem findings**	Sow death with low body condition score * (N = 26)	Sow death with normal/higher body condition score * (N = 97)	***p*-value**	**OR (CI)**
Septic locomotor disorder				
Present (N = 31)	20	11	<0.001	24.88 (7.71–93.74)
Absent (N = 92)	6	86		
Genital disorders				
Present (N = 37)	6	31	0.198	0.56 (0.16– 1.63)
Absent (N = 86)	22	64		
Urinary disorders				
Present (N = 44)	5	39	0.145	0.35 (0.09–1.08)
Absent (N = 79)	21	58		
Respiratory disorders				
Present (N = 78)	17	61	0.493	1.11 (0.41–3.14)
Absent (N = 45)	9	36		
Gastric ulcers				
Present (n = 30)	9	21	0.262	1.90 (0.65–5.33)
Absent (n = 93)	17	76		
**Post-mortem findings**	Sow death with higher body condition score * (N = 26)	Sow death with normal/low body condition score * (N = 97)	***p*-value**	**OR (CI)**
Heart failure				
Present (N = 15)	7	8	0.008	4.03 (1.10–14.57)
Absent (N = 108)	19	89		
**Post-mortem findings**	Sow death with moderate and severe claws’ injuries * **(N = 82)**	**Sow death with discrete or no claws’ injuries** **(N = 41)**	***p*-value**	**OR (CI)**
Septic locomotor disorders				
Present (N = 33)	28	5	0.015	3.69 (1.24–13.42)
Absent (N = 90)	54	36		
**Post-mortem findings**	**Sows with cystitis** **(N = 40)**	**Sows without cystitis** **(N = 83)**	***p*-value**	**OR (CI)**
Pyelonephritis				
Present (N = 15)	12	3	<0.001	11.16 (2.74–66.24)
Absent (N = 108)	28	80		
**Post-mortem findings**	**Sows exposed to manual obstetric intervention during farrowing (N = 16)**	**Sows not exposed to manual obstetric intervention during farrowing (N = 107)**	***p*-value**	**OR (CI)**
Metritis				
Present (N = 24)	10	14	<0.001	10.73 (3.00–42.22)
Absent (N = 99)	6	93		
**Post-mortem findings**	**Euthanasia (n = 23)**	**Spontaneous** **death (n = 100)**	***p*-value**	**OR (CI)**
Locomotor disorders				
Present (N = 36)	17	19	0.041	12.18 (3.95–43.02)
Absent (N = 90)	6	84		

* Low body score condition: BSC ≤ 2.5; high body score condition: BSC ≥ 3.5; peripartum: 7 days prior to farrowing and 7 days after farrowing; P-value: obtained by generalized mixed models.

**Table 2:** Post-mortem findings in spontaneous death and euthanized females.

System	Pathological Findings	Spontaneous Death N (%)	Euthanized Sows N (%)
**Locomotor**	Arthritis	11 (11)	9 (39.1)
	Suppurative myositis	9 (9)	7 (30.4)
	Fractures and non-infectious locomotor lesion	2 (2)	4 (17.4)
	Osteomyelitis	1 (1)	2 (8.7)
**Cardiovascular**	Heart failure	15 (15)	0
	Asphyxia/accident	3 (3)	0
**Gastrointestinal**	Gastric ulcer	26 (26)	4 (17.4)
	Hepatic lobe torsion and/or rupture	7 (7)	0
	Rectal prolapse	4 (4)	1 (4.34)
	Splenomegaly	3 (3)	1 (4.34)
	Spleen torsion and/or rupture	2 (2)	1 (4.34)
	Mesentery torsion and/or intestinal rupture	2 (2)	0
	Stomach dilatation	1 (1)	0
	Hepatomegaly	1 (1)	0
	Intestinal intussusception	1 (1)	0
**Genital**	Endometritis	22 (22)	2 (8.7)
	Presence of fetus or placenta retention	11 (11)	0
	Uterine prolapse	6 (6)	1 (4.34)
	Cervix rupture	6 (6)	0
	Uterine hemorrhage	3 (3)	0
	Dystocia	0 (0)	3 (13)
**Mammary**	Mastitis	4 (4)	1 (4.34)
**Respiratory**	Pleurisy	50 (50)	13 (56.5)
	Pulmonary edema (heart failure excluded)	29 (29)	2 (8.7)
	Pulmonary congestion (heart failures excluded)	23 (23)	2 (8.7)
	Pulmonary consolidation	15 (15)	1 (4.34)
	Pulmonary abscess	7 (7)	0
	Pericardium adhesion	6 (6)	0
	Pleural abscesses	1 (1)	0
**Systemic**	Peritonitis	7 (7)	0
	Polyserositis	4 (4)	0
	Splenic abscess	1 (1)	0
**Urinary**	Cystitis	35 (35)	5 (21.7)
	Pyelonephritis	14 (14)	1 (4.34)
	Hydronephrosis	2 (2)	0
	Bladder prolapse	2 (2)	0

## Data Availability

The data sets and materials are available from the corresponding author on reasonable request.

## Supplementary Materials

The following supporting information can be downloaded at https://doi.org/10.5281/zenodo.6827736. Table S1: Parity order and affected system in spontaneous death sows. Table S2: Parity order and affected system in euthanized sows. Table S3: Gestational phase and affected system in spontaneous sow deaths. Table S4: Gestational phase and affected system in euthanized sows. Table S5: Body score condition and affected system in spontaneous sow deaths. Table S6: Body score condition and affected system in euthanized sows. Table S7: Gastric ulcers’ classification. Table S8: Affected area of lungs according to pneumonia index, pleurisy, and pathogen detection. Table S9: Claw injuries’ categorization and frequencies of lesions observed. Figure S1: Bacteria isolated from arthritis cases and associations identified. Figure S2: Bacteria isolated from suppurative myositis cases and associations identified. Figure S3: Bacteria isolated from metritis cases and associations identified. Figure S4: Bacteria isolated from cystitis cases and associations identified. Figure S5: Bacteria isolated from pyelonephritis cases and associations identified.
